# Degradation insight of organophosphate pesticide chlorpyrifos through novel intermediate 2,6-dihydroxypyridine by *Arthrobacter* sp. HM01

**DOI:** 10.1186/s40643-022-00515-5

**Published:** 2022-03-27

**Authors:** Himanshu Mali, Chandni Shah, Darshan H. Patel, Ujjval Trivedi, R. B. Subramanian

**Affiliations:** 1 P. G. Department of Biosciences, UGC-Centre of Advanced Studies, Satellite Campus, Sardar Patel University, Sardar Patel Maidan, Bakrol-Vadtal Road, Bakrol, 388 315 Gujarat India; 2 Charotar Institute of Paramedical Sciences, Charotar University of Science and Technology (CHARUSAT), Changa, 388421 Gujarat India

**Keywords:** Bioremediation, Pesticides/chlorpyrifos biodegradation, Organophosphate degrading enzyme, Molecular docking, 3,5,6-trichloro-2-pyridinol

## Abstract

Organophosphates (OPs) are hazardous pesticides, but an indispensable part of modern agriculture; collaterally contaminating agricultural soil and surrounding water. They have raised serious food safety and environmental toxicity that adversely affect the terrestrial and aquatic ecosystems and therefore, it become essential to develop a rapid bioremediation technique for restoring the pristine environment. A newly OPs degrading *Arthrobacter* sp. HM01 was isolated from pesticide-contaminated soil and identified by a ribotyping (16S rRNA) method. Genus *Arthrobacter* has not been previously reported in chlorpyrifos (CP) degradation, which shows 99% CP (100 mg L^−1^) degradation within 10 h in mMSM medium and also shows tolerance to a high concentration (1000 mg L^−1^) of CP. HM01 utilized a broad range of OPs pesticides and other aromatic pollutants including intermediates of CP degradation as sole carbon sources. The maximum CP degradation was obtained at pH 7 and 32 °C. During the degradation, a newly identified intermediate 2,6-dihydroxypyridine was detected through TLC/HPLC/LCMS analysis and a putative pathway was proposed for its degradation. The study also revealed that the organophosphate hydrolase (*opd*H) gene was responsible for CP degradation, and the opdH-enzyme was located intracellularly. The opdH enzyme was characterized from cell free extract for its optimum pH and temperature requirement, which was 7.0 and 50 °C, respectively. Thus, the results revealed the true potential of HM01 for OPs-bioremediation. Moreover, the strain HM01 showed the fastest rate of CP degradation, among the reported *Arthrobacter* sp.

**Graphical Abstract:**

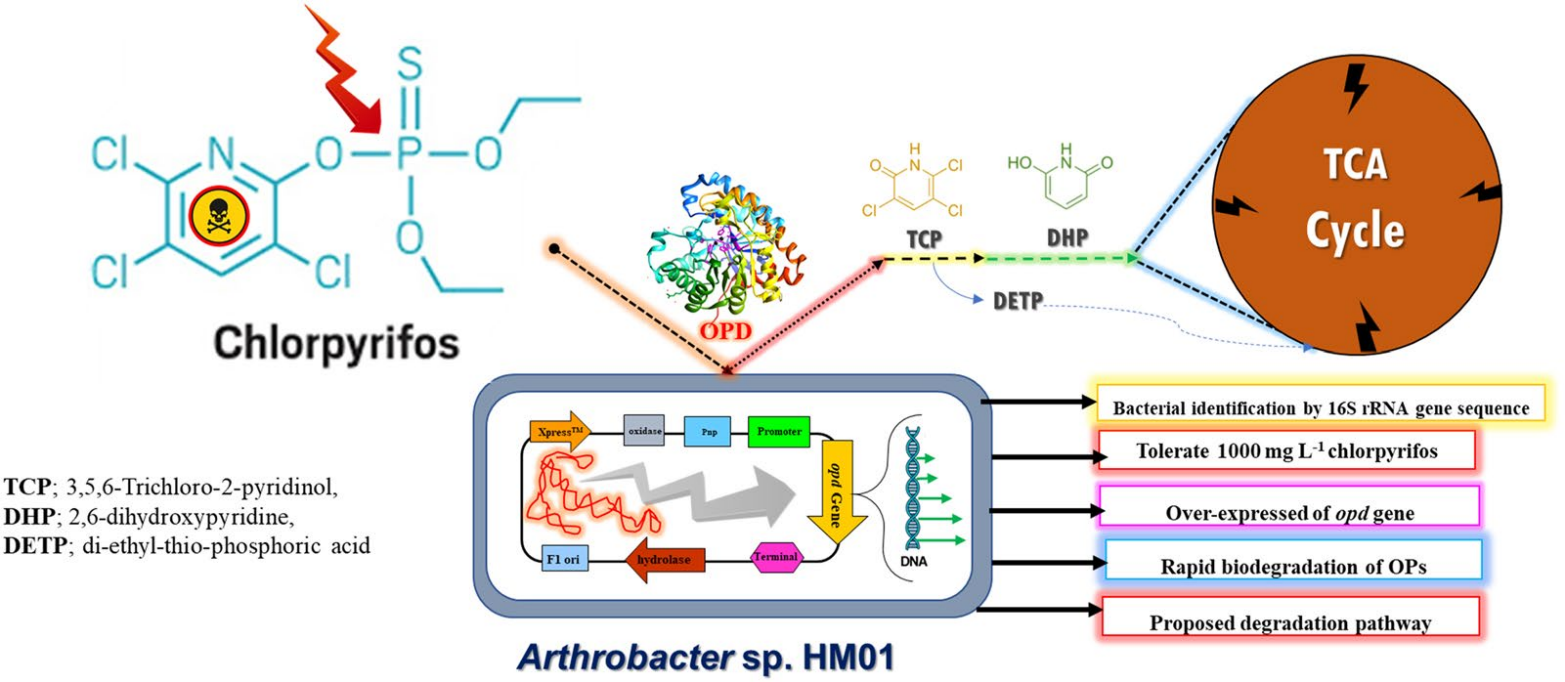

**Supplementary Information:**

The online version contains supplementary material available at 10.1186/s40643-022-00515-5.

## Introduction

International food demand is estimated to rise from 80 to 100% in the coming decades to cope with the rising human population (Foong et al. 2020). To meet this demand, farmers are using an enormous amount of agrochemicals (> 2 million) every year, including organophosphates (OPs) pesticides. They are being used to improve productivity by overcoming 40% of crop losses (equivalent to 2.5 trillion USD) incurred due to pest infestation (Peshin et al. 2009; Foong et al. 2020). However, only a small amount (approximately 1.0%) of pesticides can remove target pests, and the rest of the pesticide residues are deposited in agricultural soil, leading to leaching and hydrolysis that cause serious harm to terrestrial as well as aquatic ecosystems (Karami-Mohajeri and Abdollahi 2011; Liu et al. 2016).

The OPs pesticides were introduced in the 1950s as pest controllers and became the most commonly used insecticides in the agricultural sector in recent decades because of their high toxicity towards insects and low persistence in the soil as compared to other chlorinated pesticides such as methoxychlor (Foong et al. 2020). Although OPs are relatively less persistent in the environment and less toxic towards non-targeted animals such as birds, fish, etc., exploitation of these compounds has significantly increased in the past few decades, resulting in contamination of the food web (Intisar et al. 2022). The OPs residues were detected in surrounding environment including, agriculture crops/fruits, pond/tube-well water and many more are well documented in literature (Bose et al. 2021).

Chlorpyrifos (CP) is a toxic OPs pesticide (class II) that is substantially used in the agricultural sector for crop protection (cotton, brinjal, chillies and citrus fruits), horticulture, forestry, households and also in public health and pest management (Foong et al. 2020). CP is absorbed by different routes such as inhalation, dermal absorption and ingestion (Karami-Mohajeri and Abdollahi 2011). OPs pesticides bind irreversibly to acetylcholinesterase enzyme (AChE) of the central and peripheral nervous system (Bhatt et al. 2021). Several studies have reported that CP has adverse effects on sperm activity and hepatic and renal systems. Prenatal exposure is strongly related to low birth weight, small head size, and delayed brain development or neurological disorder in children (Andreadis et al. 2014).

The shelf-life of CP in the soil is 60–120 days, but it may recalcitrate up to 365 days depending on the environmental factors such as pH, temperature, and moisture in the soil (Singh and Walker 2006). The biotic and abiotic factors work synergistically for CP degradation in the soil. USEPA (Environmental protection agency) classified 3,5,6-trichloro-2-pyridinol (TCP) as a toxic and persistent metabolite of CP pesticides whose shelf-life was reported to be 60–180 days in agricultural soil (Rayu et al. 2017). The occurrence of three chloride (Cl_3_) atoms on the aromatic ring renders TCP resistance to microbial degradation. TCP has greater persistence and higher water solubility than CP (Bhende et al. 2021); it may accumulate in the soil or leach into the surface or ground waters because of its high mobility. It may contaminate the aquatic ecosystem or may enter into the food chain, resulting in high eco-toxicological risks (Watts 2012).

Several remediation techniques have been reported to remove such toxic pollutants from the environment to restore the pristine ecosystems. Among the proposed techniques, microbial-mediated remediation is one of the most accepted approaches towards environmental contaminants. This approach is easy to use, environmentally friendly and cost-effective (Huang et al. 2021). The most critical step in microbial bioremediation of pesticides, is the selection of microbes with the right metabolic pathway for remediation of pesticides and their degradation products such as TCP from the agricultural soil and water. Previously it has been reported that, pesticides like CP was resistant to microbial biodegradation, possibly due to the antimicrobial activity of TCP (Huang et al. 2021; Bose et al. 2021). But, later studies reported the efficient degradation of CP by the microbes, most of them were bacterial species belonging to the different genera such as *Enterobacter* (Singh and Walker 2006), *Bacillus* (Li et al. 2010), *Klebsiella* and *Pseudomonas* (Chawla et al. 2013). The main degradation products of CP were reported as TCP and DETP (di-ethyl-thio-phosphoric acid) (Kumar et al. 2018; Farhan et al. 2021). However, most of the bacterial strains can degrade only CP but not the TCP (Aswathi et al. 2019). Recently, few bacteria have been reported to degrade CP and TCP simultaneously (Fang et al. 2019). Therefore, it is important to select the bacterial strain that can simultaneously degrade both CP and TCP.

Most of the studies reported that CP degradation occurs between 2 and 28 days and the complete degradation of CP (50 mg L^−1^) was reported by *Stenotrophomonas* sp. after 28 h (Deng et al. 2015).

Recent study demonstrated that, the *Azotobacter* sp. ATCC 12837 (nitrogen fixation bacteria) degraded high concentration of CP (500 mg L^−1^) under high oxygen consumption rate, without affecting PGPR trait (Conde-Avila et al. 2021). Apart from the pure culture, several bacterial consortia-based CP degradations was also reported (Feng et al. 2017; Elshikh et al. 2022). Feng et al. isolated 5 endophytic bacterial species and developed consortium including *Pseudomonas* sp. RRA, *Bacillus* sp. RRB, *Sphingobacterium* sp. RSA, *Stenotrophomonas* sp. RSB and *Curtobacterium* sp. RSC, from the CP contaminated rice field and degraded CP (90%, 5 mg L^−1^). And also developed the protocol to mark bacterial consortium with green fluorescent protein (gfp) to track the CP degradation inside the rice plant Similarly, Elshikh et al. (2022) formulated the bacterial consortium (*Bacillus* sp. CP6 and *Klebsiella* sp. CP19) to enhance CP degradation in soil by optimizing various parameters and it could also survive in antibiotic containing environment. Study suggested the potential of consortium in CP degradation.

Similarly, several studies have showed that, besides microbes, the microbial enzymes (such as esterase enzymes from Pseudomonas sp. C11 and, hydrolase enzyme from Arthrobacter sp. HM01) are also directly use to remediate pesticides (OPs, pyrethroids and carbamide) in the environment (Bhatt et al. 2021; Mali et al. 2022a). Microbes have specific enzymes that play an essential role in pesticide degradation (Fan et al. 2018). Few well-reported OPs degrading enzymes are methyl parathion hydrolase (MPH), OP degrading enzymes (opd), OP acid anhydrolase (OPAA) and OPs hydrolase (OPH) also known as aryl-dialkyl-phosphatases or phosphotriesterases (Bhatt et al. 2021). All these enzymes belong to the family of the metallohydrolases superfamily. These enzymes cleave various ester bonds (P–O, P–S, P–C, and P–F) of OPs pesticides (CP and others) and nerve agents (Kumar et al. 2018; Mali et al. 2022c). The microbial remediation systems used for decontamination of toxic pollutants from the contaminated environments require understanding physiological, biochemical and ecological mechanisms of the pesticide degrading microorganisms.

Therefore, in this study OPs degrading bacteria were isolated from agricultural fields, having a long history of using OPs pesticides. The potent bacterium was characterized for its degradation potential which was further enhanced by optimizing a few of the abiotic and biotic parameters. The degradation profile of chlorpyrifos was studied along with the characterization of the organophosphate hydrolase (opdH) enzyme, catalyzing the first step in the chlorpyrifos degradation pathway.

## Materials and methods

### Reagents, pesticides and media

All chemicals, reagents, pesticides and standard intermediate (TCP) used in the study were of the analytical grade and purchased from Sigma Aldrich, USA. Molecular biology grade chemicals were obtained from NEB, USA and Takara, Japan. List of pesticides used in the study is mentioned in Additional file 1.

Modified mineral salt media (mMSM) (MgSO_4_.7H_2_O, 0.25 g; FeSO_4_.7H_2_O, 0.005 g; CuSO_4_, 0.01 g; KH_2_PO_4_, 7.5 g; K_2_HPO_4_, 1.25 g of; NH_4_NO_3_ 1.0 g; Ca(NO_3_)_2_.2H_2_O, 0.04 g; pH 7.0, (adjusted by 0.1 N NaOH/HCl) was used in for bacterial growth and pesticide degradation study.

### Soil sample collection

Soil samples were collected from three distinct agricultural farms: Rampura village (22°35′05.4′′N 72°54′54.5′′E), Savli village (22°24′20.8′′N 72°50′44.9′′E) and Vadtal village (22°35′25.8′′N 72°51′59.3′′E) cultivating chilli, bringle, cotton and fennel in the state of Gujarat. All fields have a long history of pesticide [chlorpyrifos (CP), malathion, methyl-parathion] application. The sub-surface (5–15 cm) soils samples were collected from three distinct sites from each field.

### Screening, isolation and identification of *Arthrobacter* sp. HM01

One gram of soil sample was suspended in 200 mL of mMSM broth, supplemented with CP and methyl parathion (50 mg L^−1^ each) as carbon sources and incubated under shaking conditions at 30 ± 2 °C for 14 days. At every 24 h, the master culture was serially diluted and spread on mMSM media containing CP and methyl parathion (50 mg L^−1^) and incubated at 30 ± 2 °C for 2–3 days. The distinctly isolated colonies were further screened on mMSM media containing increasing concentrations of CP and methyl parathion (i.e., 50, 70, 100, 200, 500 and 1000 mg L^−1^).

Genomic DNA from selected bacteria was extracted, 16S rRNA gene was amplified and sequenced using universal primers through Sanger sequencing method (Dhameliya et al. 2020) Further, organophosphate hydrolase (*opd*H) gene was isolated from *Arthrobacter* sp. HM01, using gene-specific degenerate primers (Mali et al. 2022a). The amplified (~ 800 bp) gene products were sequenced using Sanger sequencing method.

### Localization of OPs degrading opdH enzyme

The localization of opdH enzyme in HM01 strain was performed following the procedure described by Deng et al. (2015), with minor modification as described in the Additional file 1.

The enzyme assay was performed as described in Zhongli et al. (2001), with modification. Brifely, the reaction system of 1 mL consist of 100 mg L^−1^ of substrate (ethyl-paraoxon), 900 µL of phosphate-saline buffer (pH 7.0), 50 µL cell-lysate, assay system was incubated 55 °C for 10 min, and absorption was measured at 410 nm for the release of p-nitrophenol (PNP). The specific enzyme activity was defined as the amount of opdH enzyme required to produce 1 µmol min^−1^ of PNP from paraoxon under optimal assay conditions per mg of protein. The protein concentration of all the fractions was estimated by the Bradford method (Kruger 2009).

### Enzyme characterization

To study the effect of temperature and pH on opdH enzyme of *Arthrobacter* sp. HM01, the enzyme reaction (opdH enzyme with 100 mg L^−1^ ethyl-paraoxon as substrate) was incubated at range of temperature 10–80 °C at 10 °C interval. The effect of pH on opdH enzyme was checked by incubating in different pH buffer, from 1 to 10 (pH 2–3: glycine–HCl buffer, pH 3–5: sodium acetate buffer, pH 6–8: sodium phosphate buffer, pH 8–12: glycine NaOH buffer).

### Biodegradation analysis

#### Inoculum preparation

The single colony of HM01 was grown at 37 °C under shaking conditions (150 rpm) in an mMSM medium containing CP (100 mg L^−1^) as a sole source of carbon. The overnight grown cells were harvested at 6800 × g for 8 min at 4 °C. The cell pellet was twice washed with sterile saline solution (1% NaCl) and finally resuspended in sterile mMSM medium to have ~A_600_ of 0.8, and used as inoculum in further studies.

#### Initial characterization of HM01

The degradation potential of HM01 was initially characterized for its pH (3–10) and temperature (25–45 °C) requirement, tolerance to substrate concentration (CP, 20–1000 mg L^−1^). The degradation study was performed at 37 °C (except for temperature profile) under shaking condition (150 rpm) in mMSM medium containing CP (100 mg L^−1^, except for substrate concentration) as a sole source of carbon. The substrate usage profile of HM01 was also studied with stereo-chemically different 13 OPs compounds, 4 substituted mononuclear aromatic compounds and a metabolic intermediate of CP degradation, 3,5,6-trichloro-2-pyridinol (organo-heterocyclic compound), in mMSM medium supplemented with 100 mg L^−1^ of each compound separately, following plate assay method.

To study the rate of degradation of CP, residual pesticide and degraded intermediates from the entire medium (100 mL) was solvent extracted using ethyl acetate (100 mL) (at an interval of 2 h till 30 h) and concentrated using rota-evaporator (Heidolph Instruments GmbH & Co. KG, Germany) following standard procedure. The CP degradation was measured using HPLC as mention in “Statistical analysis” section. The bacterial growth was measured at 600 nm.

#### Degradation profile

The CP compound degradation profile was studied using TLC, HPLC and LCMS. Overnight grown culture (as mentioned above) was inoculated in fresh mMSM medium containing 100 mg L^−1^ CP and incubated under optimized conditions. The samples (100 mL) were withdrawn at regular interval of 2 h (till 30 h) and intermediates were extracted as mentioned above. The degraded intermediates were initially analyzed by TLC and further it was assessed using HPLC as mentioned and LCMS.

#### Analytic techniques

Thin layer chromatography (TLC) was performed on Silica gel 60 F_254_ plates (Merck Millipore, Germany), activated in HPLC-grade methanol and air-dried, prior to experiments. The CP and its degraded products were resolved using solvent system, hexane:acetone (4:1, v/v) and bands were visualized under the TLC scanner at different wavelengths (254 and 354 nm).

The high-performance liquid chromatography (HPLC) was performed using LC-20AD UFLC (Shimadzu, Japan), equipped with a C18 column (0.45 × 15 cm) and PDA detector (254 nm). The CP and degraded intermediates were resolved using isocratic mobile phase (methanol:water:: 70:30), with a flow rate of (1.0 mL min^−1^) and injection volume of 20 µL (oven temperature of 40 °C). Prior to analysis, all samples were filtered with nylon syringe filter (0.45 µm).

The degraded products were also analyzed using liquid chromatography–mass spectrometry (LCMS) (LCMS-8030 Shimadzu, Japan). The intermediates were resolved in GISS C18 column (2.1 × 150 mm) kept at 25 °C and detected using PDA detector with voltage and nebulizing gas flow maintained at 1.8 kV and 3 L/min, respectively. The sample injection volume was 20 µL following isocratic elution with a mobile phase, A (MilliQ grade water and methanol, 30:70%, v/v) and B (formic acid, 0.1%; v/v) was used for 10 min. The compound and intermediates were detected by electrospray ionization (ESI–MS) in both modes (negative and positive ion mode) with interface temperature and voltage set to 410 °C and 4.5 kV, respectively.

### Statistical analysis

The CP degradation pathway was developed in ChemDraw 19.0. The CP degradation rate in percentage was calculated as per given formula:

$$\mathrm{CP} \mathrm{degradation}=[\mathrm{residual amount in control }\left(\mathrm{uninoculated media}\right)-\mathrm{residual amount in experimental sample}]\times 100.$$

All the experiments were performed in triplicates with appropriate controls and the analytic data represented in figures were stated as mean standard deviation (± SD). All statistical analyses were performed in GraphPad Prism 9.0 and OriginPro (2018) software.

## Results and discussion

In the last few decades, we have witnessed the development of various techniques and methods in the treatment of pesticides containing ecosystems. These methods, over the years, have become more localized, losing their potential under a more heterogeneous environment. Thus, in spite of having many technologies, remediation of pesticides containing environment is still in its nascent phase. In the recent past, biological approaches have provided encouraging results in pesticide degradation. The indigenous bacteria from pesticide containing environment might have evolved the necessary enzymatic mechanisms for the metabolism of different groups of pesticides. Therefore, a competent bacterium (*Arthrobacter* sp. HM01) along with its enzymatic potential was explored to provide a rationalized approach in pesticides degradation.

### Identification and molecular characterization of HM01

During the initial isolation, 10 distinct bacteria were isolated, capable of degrading methyl parathion and chlorpyrifos (CP) in mMSM medium. Based on the higher growth rate of strain HM01 in the presence of CP in mMSM medium along with its rapid degradation, the further studies were performed using HM01 and CP. The HM01 was identified as *Arthrobacter* sp. using 16S rRNA gene sequence (Gene Accession No.: MT079332) showing nearly 99 % sequence similarity with *Pseudomonas* sp. (reclassified as *Arthrobacter* sp.). Several bacterial species have been reported to utilize OPs (CP) pesticides as a sole source of carbon and energy, such as *Flavobacterium* sp., *Pseudomonas* sp., *Agrobacterium* sp., *Bacillus* spp*.* and *Burkholderia* sp. (Iyer et al. 2013).

### Multi-substrate study of HM01

**Fig. 1:**
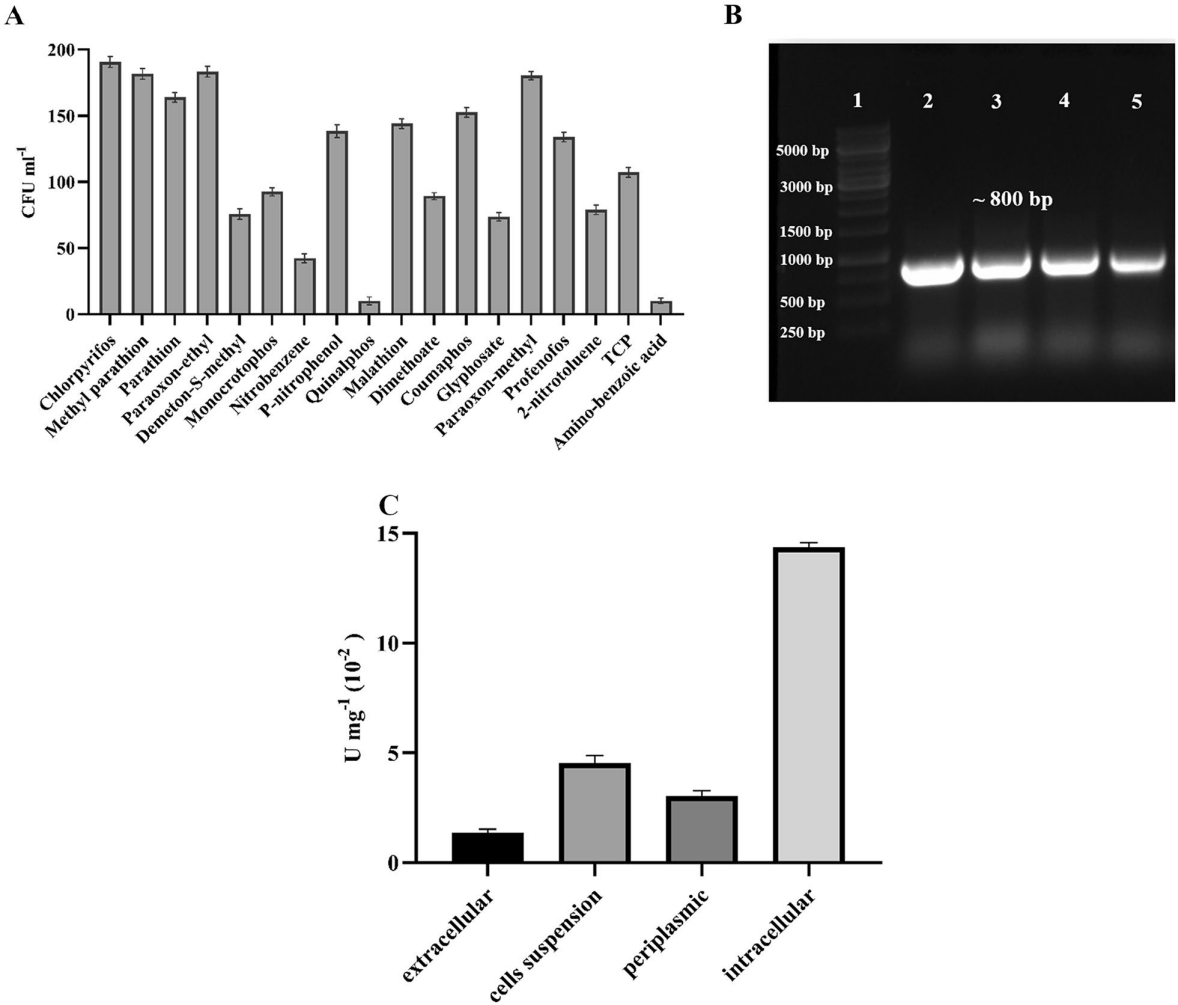
**A** Multi-substrate analysis of *Arthrobacter* sp. HM01, **B** amplicon of *opd*H gene on 1% agarose gel with 1 kb DNA ladder, lane 1. DNA ladder (1 kb), lanes 2–5 are *opd*H amplicons of approximately 800 bp, **C** localization of opdH enzyme in isolate HM01

The HM01 grew rapidly on mMSM media containing various OPs pesticides and aromatic compounds (100 mg L^−1^) as a sole carbon sources as shown in Fig. 1A. Besides OPs, it also degraded 3,5,6-trichloro-2-pyridinol (TCP, major intermediate of CP degradation) and other aromatic compounds (benzene and toluene), metabolizing as carbon source. Other phosphotriesters compounds like parathion and paraoxon were also degraded by HM01 by cleaving tri-ester linkage of O-P bonds. Therefore, HM01 not only degraded various OPs, but it has also degraded the toxic intermediate TCP, which possibly indicates that the toxicity of CP and its intermediates was decreased by HM01. *Arthrobacter* sp. has been previously reported in aromatic compounds (toluene, nicotine and benzene) degradation along with few pesticides (Iyer 2013).

### Molecular identification of OPs degrading gene from HM01

Using the gene-specific primers and optimized PCR conditions as described by Mali et al. (2022a), OPs hydrolase (*opd*H) gene was isolated from *Arthrobacter* sp. HM01. The molecular size of *opd*H amplicon was ~800 bp, (GenBank Accession No. MW413755) (Fig. 1B), which showed 93%, 90% and 80% gene sequence homology with the reported *opd*H gene from different bacterial species, *Pseudomonas putida* (KC189957), *Sphingobium fuliginis* ATCC 27551 (CP041020), and *Brevundimonas diminuta* (HQ839670), respectively (Thakur et al. 2019).

OPs degrading enzymes play a vital role in degrading various toxic OPs pollutants from the contaminated environment. OPs hydrolyzing enzymes were reported to degrade various OPs pesticides such as paraoxon, methyl parathion and CP (Iyer et al. 2013; Thakur et al. 2019; Mali et al. 2022b). Abraham and Silambarasan (2016) also reported the methyl parathion hydrolase gene of *Ochrobactrum* sp. JAS2, played a key role in degradation of CP and TCP. Similarly, Fang et al. (2019) demonstrated the role of three genes, (a) organophosphorus hydrolase (*opd*B) for CP, (b) NADPH: flavin reductase (*fre*), (c) 2,4,6- trichlorophenol monooxygenase (*tcp*A) for TCP involved in removal of CP and TCP. Similarly, different types of OPs degrading genes and their expression patterns were reported in a diverse group of organisms such as *Flavobacterium* spp., *Pseudomonas* sp*., Agrobacterium* sp., *Bacillus* spp*.* and *Burkholderia* sp. (Kumar et al. 2018; Thakur et al. 2019). OPs degrading enzymes belong to the hydrolase family, which catalyzes the hydrolysis of compounds containing various bonds such as O–P, C–P, P–S, and P–F (Thakur et al. 2019).

### Molecular docking results

To understand the enzyme–substrate interaction, in silico modeling and molecular docking were performed. To observed the molecular interaction between CP and opdH, tools like SWISS-MODEL, AutoDock Vina 1.2, Biovia Discovery studio 2.0, were used and docking results revealed that the most active or conserved amino acids of opdH enzyme was His-13, His-15, Trp-89, Lys-127, His-159, His-188, Asp-191, Hsi-212, His-215, and Asp-259, which prominently interacted with CP (Additional file 1: Fig. S1). Due to the presence of three chlorine (Cl) atoms in CP pesticide, the majority of interactions are alkyl and pi-alkyl. The Lys-127, His-188 residues of the opdH enzyme might have interacted with CP by traditional hydrogen bond (H-bond) and C–H bond, respectively. Among all the interactions, only His-159 has directly interacted with the benzene ring of CP via alkyl or π-alkyl. Similarly, the role of lysine a conserved amino acid of phosphotriesterase (PTE) of *Pseudomonas diminuta*, in OPs pesticides degradation was also reported by Bigley and Raushel (2013).

### Localization of opdH enzyme

The isolate HM01 has an OPs hydrolase (*opd*H) gene involved in the CP biodegradation. It was also tested for paraoxon, since the opdH enzyme can degrade wide range of substrates, including CP, paraoxon, malathion and quinalphos (Singh and Walker 2006; Iyer et al. 2013). Enzymatic hydrolysis of paraoxon produces p-nitrophenol, which could be determined by colourimetry (Munnecke and Hsieh 1976). The maximum activity (14.4 × 10^–2^ U mg^−1^) was observed in the intracellular fractions, while the lowest (1.37 × 10^–2^ U mg^−1^) was observed in the extracellular fractions, as shown in Fig. 1C. Based on the crude enzyme activities, each fraction could be arranged as follows; intracellular fraction > extracellular > periplasmic > whole cells. Therefore, with the available results, the opdH enzyme has to be intracellular. Similar results were also reported earlier in other bacterial strains such as *Plesiomonas* sp. M-6 (Zhongli et al. 2001), *Stenotrophomonas* sp. G1(Deng et al. 2015), and *Pseudomonas nitroreducens* AR-3 (Aswathi et al. 2019), while the periplasmic OPs hydrolase enzyme was reported in *Brevundimonas diminuta* (Gorla et al. 2009).

### Biochemical characterization of opdH enzyme

#### Effect of pH on opdH enzyme activity

**Fig. 2:**
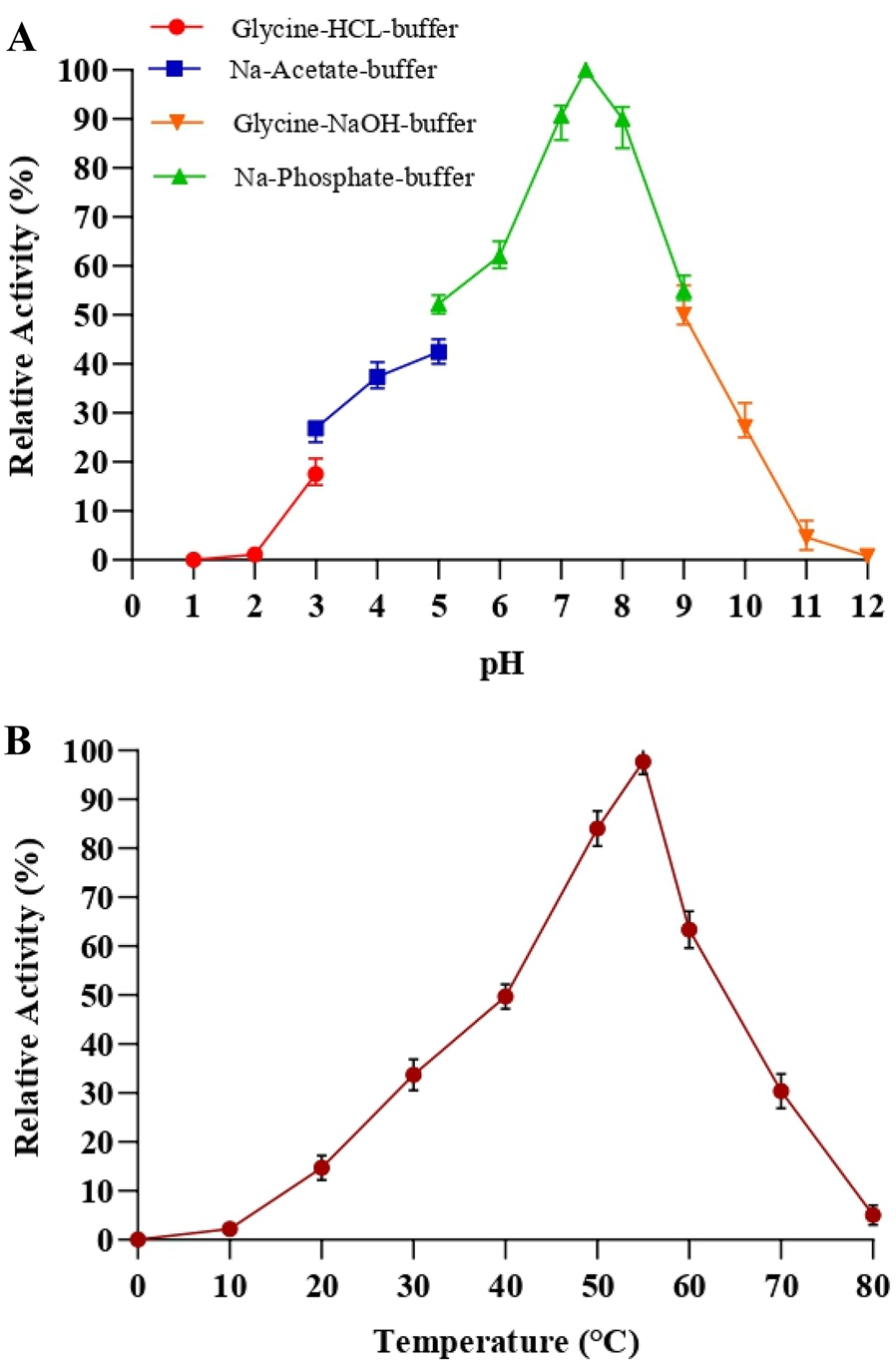
Biochemical profiling of opdH enzyme of *Arthrobacter* sp. HM01, **A** pH profiling of opdH enzyme, **B** temperature profiling of opdH enzyme

The effect of pH on the relative specific activity of the opdH enzyme was investigated in the range of pH 2.0–12.0. The pH profile of opdH revealed that the maximum relative specific activity was observed at pH 7.0, while the lowest was found at pH 3.0 and 10.0, as shown in Fig. 2A. The results suggested that the enzyme activity was notably influenced under the neutral to alkaline pH as compared to an acidic solution. The opdH enzyme of *Arthrobacter* sp. HM01 was completely inactive under extreme pH conditions, acidic (< pH 3), and alkaline (pH < 10). However, the opdH could retain > 50% activity at pH 5.0 and 9.0.

Several reports suggested that the OPs degrading enzymes such as methyl parathion hydrolase from *Azohydromonas australica* (Zhao et al. 2021), organophosphorus hydrolase from *Pseudomonas* sp. C2-1 (Chu et al. 2006), are stable and shows the highest activity under neutral to alkane conditions. Although, the OPs degrading esterase enzyme from *Geobacillius* sp. TK4 exhibited high activity at pH 3.0–9.0, however, the enzyme lost more than 80% of activity at pH 9.0 and above (Yildirim et al. 2009). Comparably, our opdH enzyme retained more than 50% activity in pH range 5.0–10.0, which was higher than the reported enzyme-like esterase of *Geobacillius* sp. TK4 (Yildirim et al. 2009).

#### Effect of temperature on opdH enzyme activity

The effect of temperature on opdH enzyme activity was investigated from 10 to 80 °C, as shown in Fig. 2B. The opdH enzyme of *Arthrobacter* sp. HM01 showed highest relative activity at 55 °C and lowest was observed below 20 °C and above 70 °C. However, opdH enzyme could retain more than 50% activity in the range of 30–60 °C. The result suggested that the opdH enzyme have optimal temperature of 55 °C and sensitive to higher temperature (> 60 °C) and sharply decreased the activity above 60 °C. Similar results were also observed in recombinant organophosphate hydrolase (ropdH) enzyme of *Arthrobacter* sp. HM01 (Mali et al. 2022b). The most of OPs degrading microbial enzymes have optimal temperature from 30 to 50 °C. The opdH enzyme from *Arthrobacter* sp. HM01 have high temperature tolerance as compared to reported OPs degrading enzymes. For instance, methyl parathion hydrolase enzymes from *Ochrobactrum* sp. M231, *Pseudomonas* sp. WBC-3 and organophosphorus hydrolase (opdD) enzyme from *Lactobacillus* sp. WCP904, have optimal temperature of 37, 40 and 30 °C, respectively (Tian et al. 2010; Haque et al. 2018; Li et al. 2018). The chlorpyrifos hydrolase was isolated and characterized from *Cladosporium* sp. Hu-01 and the optimized temperature and pH were 40 °C and 6.5, respectively (Gao et al. 2012). Also, enzyme was sensitive to high temperature (> 50 °C) and pH (> 8.0), which was comparably lower than opdH enzyme of *Arthrobacter* sp. HM01.

### Effect of environmental factor on CP degradation

**Fig. 3:**
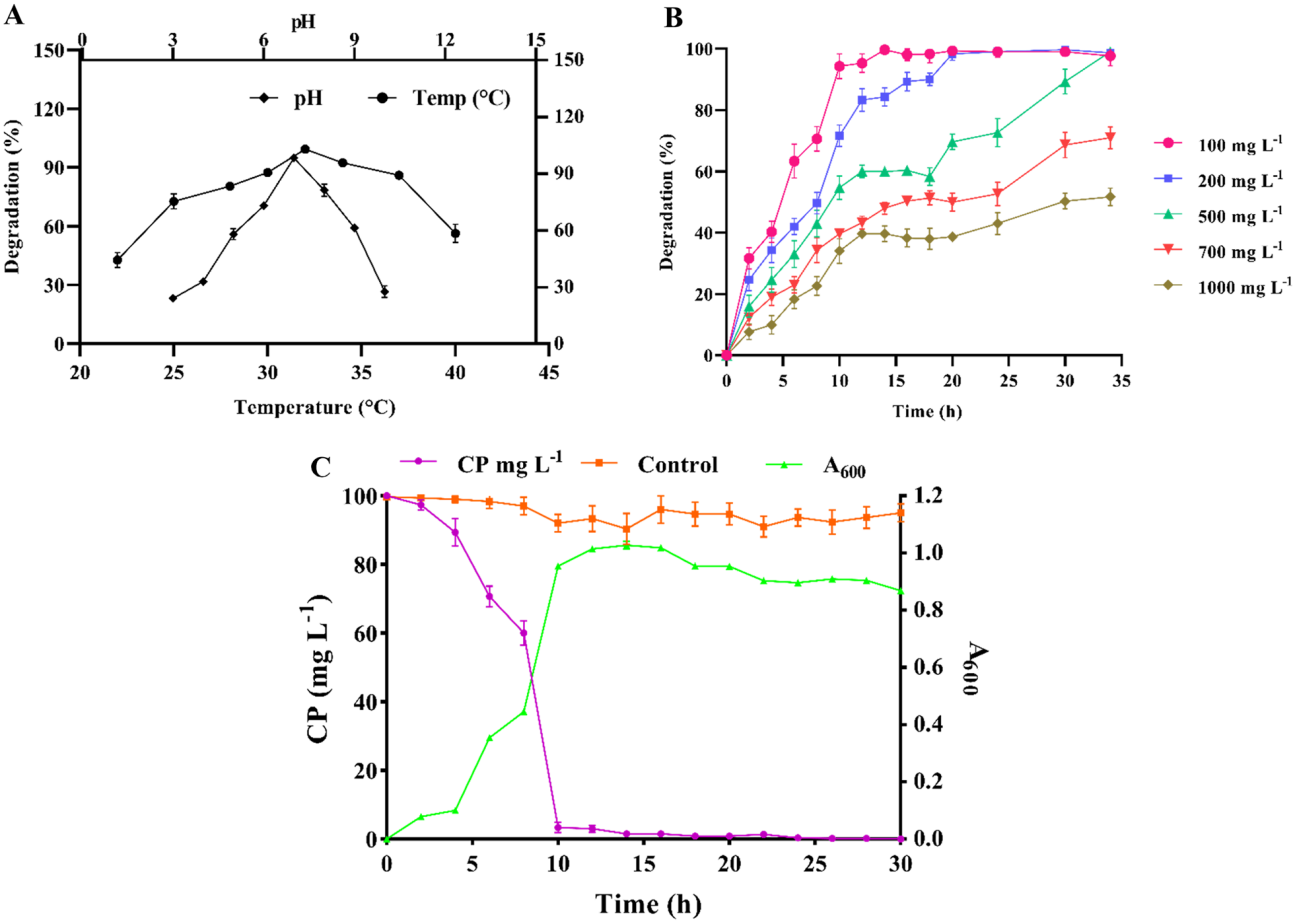
Effect of environmental factor on CP degradation by *Arthrobacter* sp. HM01. **A** Effect of pH and temperature, **B** tolerance of HM01 towards CP concentration from 100 to 1000 mg L^−1^, **C** degradation profiling of CP by HM01 (100 mg L^−1^)

Critical parameters including pH, temperature and different concentrations of CP were optimized for rapid biodegradation of OPs compound by HM01. As shown in Fig. 3A, the maximum rate of CP degradation (≥ 99%) was observed at neutral pH 7 and 32 °C, while at 22 °C the degradation rate was 47% and at 40 °C it was 58%. The results clearly indicated that under acidic and alkaline conditions was less favorable for CP degradation. Similarly, Farhan et al. (2021), isolated *Bacillus* sp. Ct3 from CP contaminated cotton soil, which has degraded 88% of CP (125 mg L^−1^) within 8 days. Several mathematical models (Plackett–Burman design, Central composite design, Michaelis–Menten model) were used to optimize degradation profiling of *Bacillus* sp. Ct3 for CP and optimized pH and temperature were 8 and 35 °C (Farhan et al. 2021).

Isolate HM01 was also grown at different concentrations of CP (100–1000 mg L^−1^) in mMSM medium under optimized conditions. Samples were harvested periodically for monitoring the progress of CP degradation using  HPLC. HM01 degraded ≥ 99% of 100, 200, and 500 mg L^−1^ of CP within 10, 20, and 34 h, respectively. Also, HM01 not only tolerated a high concentration of CP (1000 mg L^−1^), but also degraded 50% within 34 h, as shown in Fig. 3B. Considering the results, the metabolic activity of HM01 was not completely suppressed by catabolic repression under high CP concentration. However, different bacterial species (*Bacillus* sp., *Pseudomonas* sp., *Achromobacter* sp., and *Ochrobactrum* sp.) were reported to degrade CP (100 mg L^−1^) in 1–28 days (Table 1) (Iyer et al. 2013; Akbar and Sultan 2016; Foong et al. 2020).

**Table 1:** CP degradation rate of isolate HM01 and other reported microorganisms

Sr no.	Isolated microorganisms	Initial CP concentration (mg L^−1^)	Time in days	pH	Temperature (°C)	Degradation (%)	References
1	*Arthrobacter* sp*.* HM01	100	0.5	7.0	32	99	Present study
2	*Arthrobacter* sp*.* HM01	200	0.9	7.0	32	100	Present study
3	*Arthrobacter* sp*.* HM01	500	1.5	7.0	32	100	Present study
4	*Bacillus* sp. Ct3	125	8.0	8.0	35	88	(Farhan et al. 2021)
5	*Azotobacter* sp. ATCC 12837	500	2.4	7.2	29	99	(Conde-Avila et al. 2021)
6	*Bacillus pumilus* C2A1	100	10.0	8.5	37	88	(Foong et al. 2020)
7	*Sphingobacterium* sp. C1B	42	14.0	–	20	100	(Verma et al. 2020)
8	*Cupriavidus* sp. *X1*^*T*^	100	1.0	7.0	37	92	(Fang et al. 2019)
9	*C. nantongensis.* X1^T^	200	2.0	8.0	37	100	(Shi et al. 2019)
10	*Achromobacter* sp. JCp4	100	10.0	–	30	84.4	(Akbar and Sultan 2016)
11	*Ochrobactrum* sp*.* FCp1	100	10.0	–	30	78.6	(Akbar and Sultan 2016)
12	*Bacillus* sp. BRC-HZM2	100	1.0	–	30	88	(Wu et al. 2015)
13	*Cladosporium* sp. Hu-10	50	5.0	6.5	27	90	(Chen et al. 2012)

### Biodegradation of CP by HM01

To study the biodegradation profile of CP, samples were periodically (2 h) withdrawn from the experimental flask and the concentration of CP residues was determined by HPLC. The rate of CP degradation (10 mg L^−1^ h^−1^) and proliferation of HM01 was high after 6 h of incubation (Fig. 3C). While after 6 h, the degradation rate was notably increasing and it has degraded ≥ 99% of CP within 10 h of incubation, bringing down the pesticide (CP) concentration to as low as 1 mg L^−1^. Recently it was reported that as the concentration of pesticide residues decreases in the medium, the CP degradation rate also decreases drastically (Anwar et al. 2009; Aswathi et al. 2019). Increasing the concentration of pesticides from 100 to 1000 mg L^−1^ (Fig. 3), there was minor increase in the percentage of CP degradation. Previous reports support the observation that, higher initial concentrations of CP (> 100 mg L^−1^) elevates the CP degrading enzyme levels in the bacterial cell (Yang et al. 2005). The biodegradation of CP by *Pseudomonas* sp. PS-2 from the rhizospheric soil removed complete CP (100 mg L^−1^) within 28 days (Korade and Fulekar 2009). Lu et al. (2013) reported that an incubation time of 6 h was required for  degrading CP pesticide (10 mg L^−1^) by *Cupariavidus* sp. DT-1. A similar study shows that, *Bacillus thuringiensis* sp. BRC-HZM2, degrade 88% CP at 200 mg L^−1^ concentration after 24 h of incubation under optimized conditions (Wu et al. 2015). Furthermore, the CP degradation rate of HM01 and recently reported microorganisms are comparatively described in Table 1.

#### Identification of metabolites and proposed CP degradation pathway

**Fig. 4:**
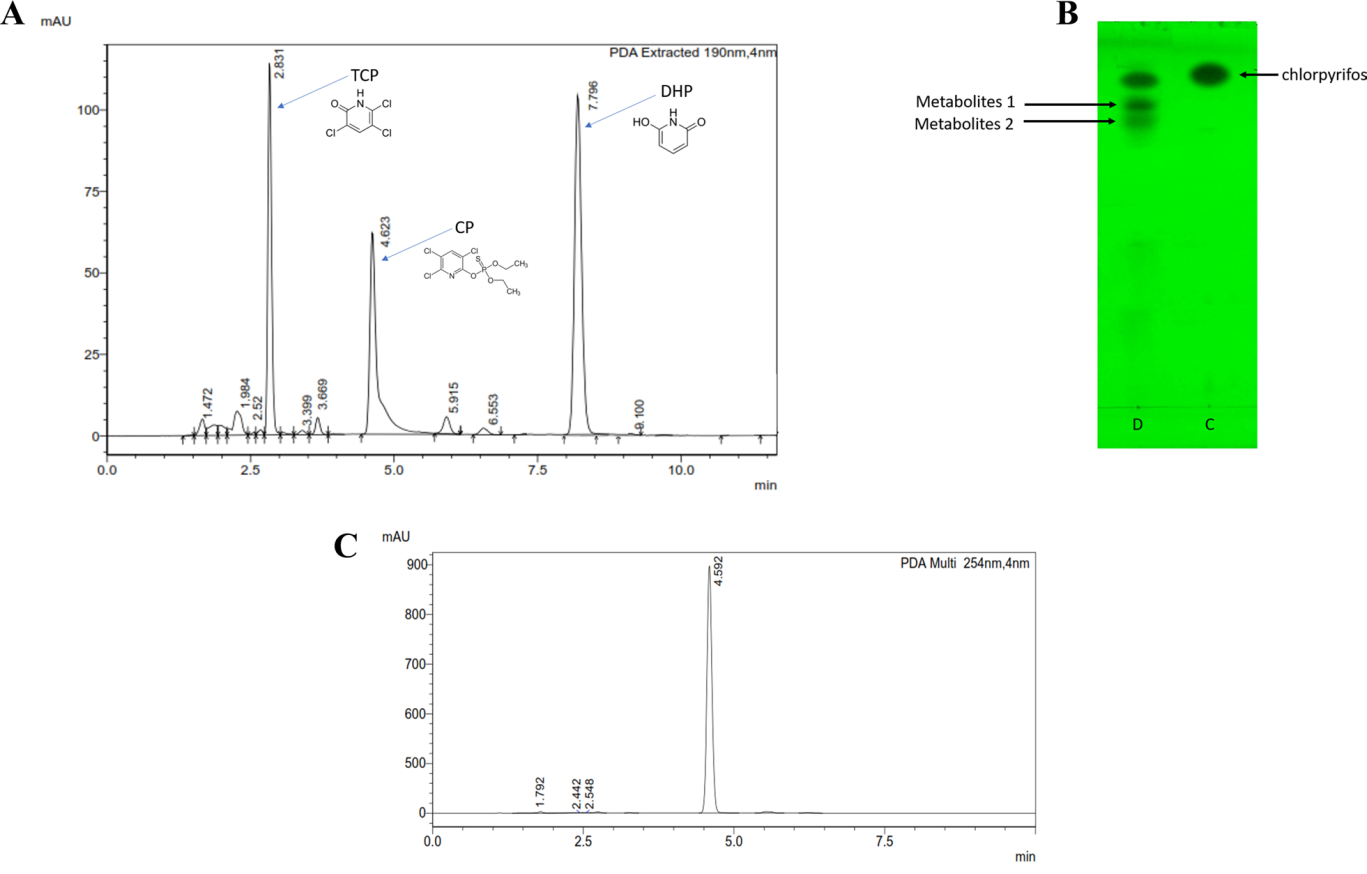
**A** HPLC profile of CP metabolites by HM01, where a sample was harvested after 10 h, **B** TLC profile of CP metabolites by HM01, where metabolite 1 and 2 are identical to TCP and DHP with CP, wherein D is degradation sample, C is control (parent compound), **C** chromatogram of CP standard

**Fig. 5:**
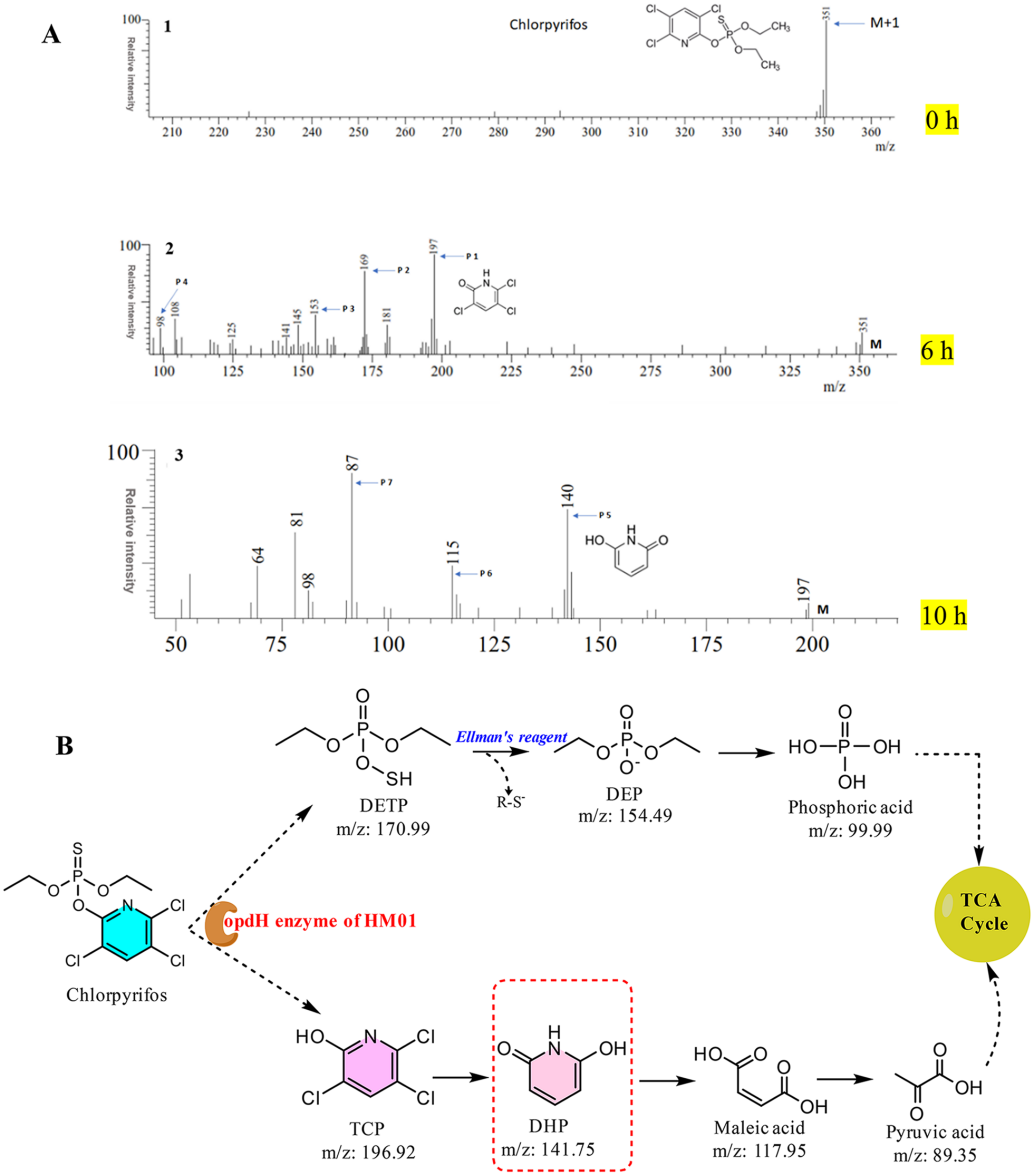
**A** Mass spectra of chlorpyrifos (CP) metabolites in the degradation process, wherein (P1) was TCP, (P2) DETP, (P3) DEP, (P4) H3PO4, (P5) DHP, (P6) *cis*-2-butenedioic acid, (P7) pyruvic acid, **B** proposed CP degradation pathway by isolate HM01, where all metabolites were sequentially arranged with *m/z* value, releasing of thiol group from DETP was measured by Ellman’s reagent, and red dotted box represents new metabolite observed in CP degradation pathway

The biodegradation of CP and its degraded products or metabolites were extracted by solvent extraction method, and analyzed and confirmed by several analytical techniques: TLC, HPLC and LCMS. Initially, CP metabolites were analyzed by TLC, which revealed the formation of two metabolites along with parent compounds. Rf values of these two compounds matched with standard TCP (0.66), 2,6-dihydroxypyridine (DHP) (0.61) and parent CP compound (0.57) as shown in Fig. 4. Relatively similar band patten of CP/TCP on TLC were also observed in CP degradation by *Lactobacillus brevis* WCP902 (Islam et al. 2010). Extracted metabolites were further analyzed by HPLC, which showed three significant peaks with different retention times: 2.80 min identical to standard TCP (Additional file 1: Fig. S2), 4.62 min identical to standard CP and 7.79 min similar to the standard DHP compound (Fig. 4A). Similar results were also observed in the earlier studies (Sachelaru et al. 2005; Verma et al. 2020). Further, CP degraded products were analyzed by LCMS. During this experiment in the initial stages of degradation, the parent (CP) compound mass spectra showed a prominent molecular ion peak at *m*/*z* 351, which matches the standard CP *m*/*z* 350 as shown in Fig. 5A. However, the sample removed after 6 and 10 h of degradation showed a major molecular ion peak (P1) at *m*/*z* 197. It was identified as 3,5,6-trichloro-2-pyridinol (TCP) by comparing with the TCP standard. The second metabolite (P2) was identified as di-ethyl-thio-phosphoric acid (DETP) at *m*/*z* 169. Moreover, its fragment ion was observed at *m*/*z* 140 and 94, which showed loss of H, 2H_5_ + C_2_H_5_ + O and C_2_H_5_. Earlier it was reported that CP transformed into TCP (P1) and DETP (P2) by the process of hydrolysis (Shi et al. 2019). Meanwhile, as metabolites P1 and P2 were unstable, they further metabolized into P3, P4, P5 and P6. Metabolite P3 was identified as di-ethyl-acid-phosphate (DEAP) when compared with standard molecular ion peak at *m*/*z* 152, and a molecular ion peak at 124 indicates loss of C_2_H_5_ and H^+^. Furthermore, releasing of thiol group from DEPT was detected by the Ellman’s reagent. The metabolite P3 further converted into P4, identified as phosphoric acid (H_3_PO_4_), matching with standard (*m*/*z* 98.0). HM01 further dechlorinated TCP to produce P5, which was identified as DHP (2,6-di-hydroxy-pyridine) with molecular peaks at *m/z* 162, 136 and 107, indicating the loss of 3Cl, O, 2C_2_O_2_ and CHON. The DHP was a newly identified intermediate during the CP degradation pathway. The discovery of P5 indicated that all the three chlorine atoms were removed step by step from the TCP. The ring-opening product was *cis*-2-butenedioic acid (P6) with a molecular peak at *m*/*z* 116. It could be further converted into pyruvic acid (P7), which was identified by the molecular ion *m*/*z* as 88 as compared to the standard *m*/*z* value. According to the KEGG (Kyoto-Encyclopedia of Genes and Genomes) database (Shi et al. 2019), metabolite P7 could enter into the TCA (tri-carboxylic acid) cycle and completely metabolize into the CO_2_ and H_2_O. Figure 5B shows the proposed CP degradation pathway by HM01. Recently, Fang et al. (2019) reported the new CP degradaing bacterium *Cupriavidus* sp. X1^T^ removed 100 mg L^−1^ CP and 20 mg L^−1^ TCP within 6.0 and 8.0 h, respectively. Also, they annotated the CP, TCP degradation genes cluster and characterized the key genes *opd*B, *fre*, and *tcp*A for degradation of CP and TCP and proposed the pathway. Additionally, the rhizospheric bacteria have plant growth promoting trait and also utilized a chemical pollutant as carbon source. Several bacterial consortia have been reported for CP degradation. Recently, Feng et al. (2017) isolated five endophytic bacteria from rice plant, as mentioned earlier and experimentally shown in vivo as well in vitro degradation of CP (5 mg L^−1^ 90% in 24 h) in the rice plant. The CP degradation is not limited to bacteria, few fungi also reported to degrade CP as carbon source, for instance, Chen et al. (2012) reported a new fungal *Cladosporium* sp. Hu-10 for degradation of CP (50 mg L^−1^) and TCP under optimized condition (26.8 °C, pH 6.5) within 5 days, which could be a potential strain for bio-control and bioremediation of CP.

For the implementation of the microbial bioremediation strategy, it is crucial to analyze the toxic nature of the intermediate metabolites produced during this process. Degradation of CP pesticides produces toxic metabolite (TCP) during biodegradation (Rayu et al. 2017; Verma et al. 2020). TCP is highly persistent in soil (60–360 days) and has greater water solubility than the parent compound (Rayu et al. 2017). Our result demonstrated that isolate HM01 not only degrades CP, but also degraded TCP into non-toxic forms (2,6-dihydroxypyridine). As shown in Fig. 1A, HM01 efficiently used toxic TCP (100 mg L^−1^) as a sole carbon source for growth. However, more specific and detailed studies are required to investigate the toxicity of each CP intermediates.

OPs degrading enzyme (PTE) acts as a regulatory or key enzyme for CP degradation, which transforms them into alcohol and acid as by-products (Shi et al. 2019; Verma et al. 2020; Mali et al. 2022c). Cleavage of the ester bond (P–O) of CP indicates the over-expression of the *opd*H gene in HM01, which leads to accelerated degradation of CP. The intermediate metabolites of CP have been arranged in a logical sequence to propose a new CP degradation pathway by HM01, as shown in Fig. 5B, because a new degradation product (2,6-dihydroxypyridine) was detected during CP degradation. This metabolite (2,6-dihydroxypyridine) was also observed in nicotine degradation by *Arthrobacter nicotinovorans* (Sachelaru et al. 2005).

Many authors have used bacteria, fungi, animal, plant, and microbial enzymes to study the bioremediation of OPs pesticides such as CP, malathion, paraoxon (insecticide), glyphosate, and coumaphos (herbicides) (Iyer et al. 2013; Kumar et al. 2018; Foong et al. 2020). They found the microbial degradation system to be more suitable and/or efficient for field application than the enzymatic degradation system because it required more specialized conditions such as specific pH and temperature. In unfavorable environmental conditions, the microbial enzyme activity might decrease. However, in the case of microbial degradation, limitations are relatively less since microbes can grow in variable or oligotrophic environmental conditions such as high/low temperature, pH, and low nutrients (Singh and Walker 2006; Verma et al. 2020). Altogether, microbial degradation could be one of the most promising, cost-effective, and environmentally friendly bioremediation approaches to remove toxic pollutants from the environment (Iyer et al. 2013; Kumar et al. 2018). Also, several studies have been reported, but were limited to the ideal lab conditions (Rayu et al. 2017; Aswathi et al. 2019; Shi et al. 2019; Verma et al. 2020).

## Conclusion

Thus, during the study, it became evident that *Arthrobacter* sp. HM01 (which was not been previously reported in OPs degradation) had a potential for OPs metabolism. Chlorpyrifos was metabolized as a sole source of carbon, which suggests the adaptation of HM01 for an oligotrophic environment. The degradation profile showed the newly identified intermediate 2,6-dihydroxy pyridine during chlorpyrifos degradation by bacteria. With the array of results obtained along with the initial catalytic mechanism of the *opd*H gene, a further study has been initiated to improve the activity and substrate specificity of the opdH enzyme using recombinant technology and protein engineering approaches for possible in situ bioremediation of OPs contaminated environment.

### Supplementary Information

**Additional file 1: Figure S1.** Molecular docking of chlorpyrifos pesticide in catalytic pocket of opdH enzyme, where conserved residues were interacted with pesticides. **Figure S2.** HPLC chromatogram of standard intermediate 3,5,6-Trichloro-2-pyridinol (TCP) of chlorpyrifos pesticide.

## Data Availability

All relevant material and data used in this study will be provided on request by the corresponding author on valid request.
