# Supplementary material for: Degradation insight of organophosphate pesticide chlorpyrifos through novel intermediate 2,6-dihydroxypyridine by Arthrobacter sp. HM01
Source: Bioresour Bioprocess. 2022 Mar 27;9(1):31. doi: 10.1186/s40643-022-00515-5 (PMC10992969; doi:10.1186/s40643-022-00515-5)
Supplement: Supplementary file 1 — Additional file 1: Figure S1. Molecular docking of chlorpyrifos pesticide in catalytic pocket of opdH enzyme, where conserved residues were interacted with pesticides. Figure S2. HPLC chromatogram of standard intermediate 3,5,6-Trichloro-2-pyridinol (TCP) of chlorpyrifos pesticide. [file 40643_2022_515_MOESM1_ESM.docx]

*Supplementary material*

**Degradation insight of organophosphate pesticide chlorpyrifos through novel intermediate 2,6-dihydroxypyridine by *Arthrobacter* sp. HM01**

Himanshu Mali^1^, Chandni Shah^1^, Darshan H. Patel^2^, Ujjval Trivedi^1^, R. B. Subramanian^1^*

P. G. Department of Biosciences, UGC-Centre of advanced studies, Satellite campus, Sardar Patel University, Sardar Patel Maidan, Bakrol-Vadtal Road, PO Box 39, Vallabh Vidyanagar, Gujarat 388 120, India

^b^ Charotar Institute of Paramedical Sciences, Charotar University ofScience and Technology, (CHARUSAT), Changa, Gujarat 388421, India

*Corresponding author:

R. B. Subramanian

^a^.P. G. Department of Biosciences, Sardar Patel University,

Sardar Patel Maidan, Vadtal Road, Bakrol 388 315, Gujarat, India

Phone: +09510371267

Email: [promat103vans@gmail.com](mailto:promat103vans@gmail.com)

**Materials and methods**

*Reagents, pesticides, and media*

Analytical grade paraoxon-ethyl (99%), chlorpyrifos (99%), parathion, malathion (99%), glyphosate (99%), Coumaphos (99%), profenofos (99%), dimethoate (99%), Dementos-s-methyl (99%), Monocrotophos (99%), and standard intermediates (3,5,6-Trichloro-2-pyridinol) (99%) purchased from Sigma Aldrich and local agrochemicals shops at 50% emulsifiable concentrate (EC).

*Molecular identification of Arthrobacter sp HM01*

The genomic DNA was isolated as per described method (Dhameliya et al., 2020) and used as a template for 16s rRNA gene amplification by PCR using universal primer; 27F forward primer (5'AGA GTT TGA TCC TGG CTC AG 3'), and 1492R reverse primer (5'ACG GCT ACC TTG TTA CGA CTT 3'). Optimized PCR parameters are as follows: initial denaturation temperature set as 95˚C at 300 seconds then 30 seconds for remaining 30 cycles, annealing at 51˚C for 30 seconds, extension at 72˚C for 90 seconds, final extension at 72˚C for 300 seconds and reaction hold at 4˚C. Amplified products were visualized on 1% agarose gel, and the same was purified from the gel by PCR clean-up kit (Macherey-Nagel™). Purified PCR products were sent for sequencing, and the obtained DNA sequences were analyzed using the NCBI BLAST tool. Phylogenetic analysis: the molecular evolutionary tree was generated using MEGA 5.05 with default settings (Kimura two-parameter distance model calculated relative distances, an un-rooted tree was constructed by the neighbor-joining method, and bootstrapped data was set to 1000 times for validation of dendrograms) (Tamura et al., 2011).

*Localization of OPs degrading OPD enzyme*

The localization of OPD enzyme in HM01 strain was performed following the procedure described by Deng et al. (2015b) (Deng et al., 2015) with minor modification. Briefly 14 h grown culture (in mMSM medium containing CP, 100 mg L^-1^) was harvested at 6800 x g at 4°C for 12 min. The supernatant was filtered through a 0.22 µm cellulose filter, and the extracellular fraction was stored at 4°C. The cell pellet was washed thrice with normal saline and resuspended in a minimum quantity of Tris-Cl buffer (0.01 M; pH 7). For intracellular enzyme localization, the resuspended cells were lysed using ultra-sonication (30 % amplitude for 20 sec ON and 5 sec OFF up to 5 min under cold condition) and cell lysate was centrifuged at 9,300 x g at 4°C for 12 min, and supernatant mixed with PMSF (phenylmethylsulfonyl fluoride) was stored at 4°C for an intracellular enzyme. For periplasmic enzyme localization, the harvested cell pellet was subjected to osmotic shock as described by Yang (Yang et al., 2005) and stored at 4°C till further use.


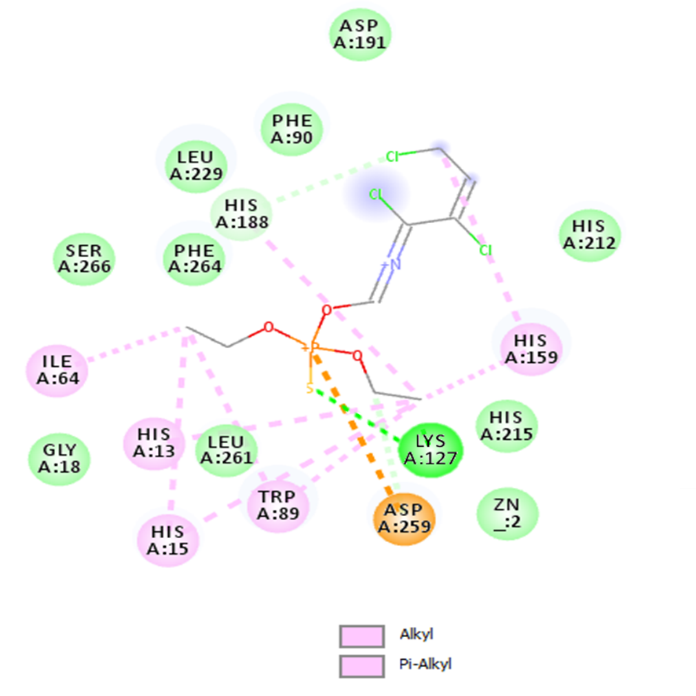

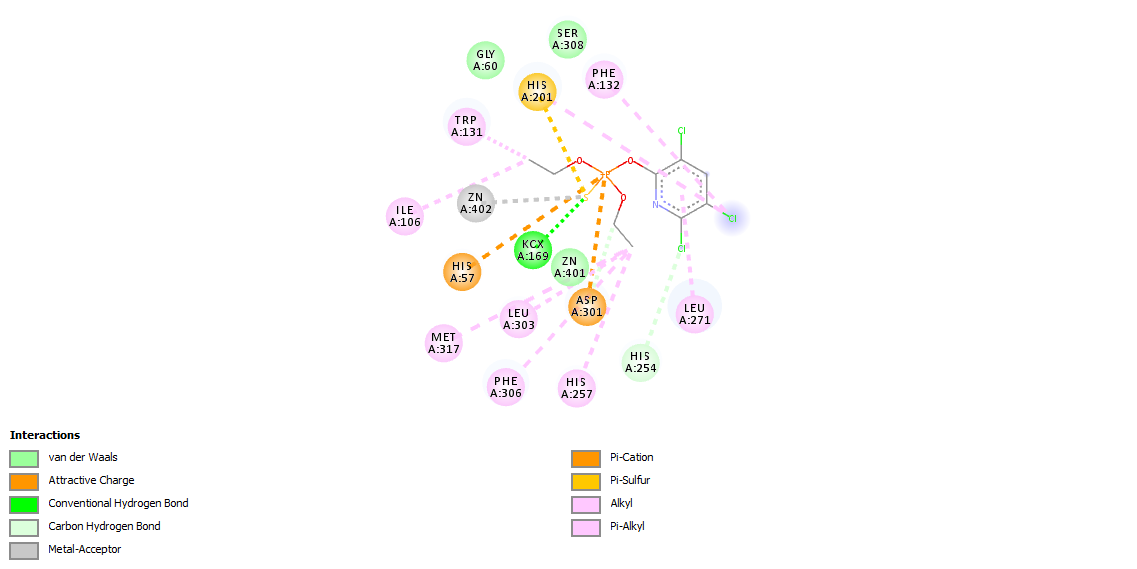

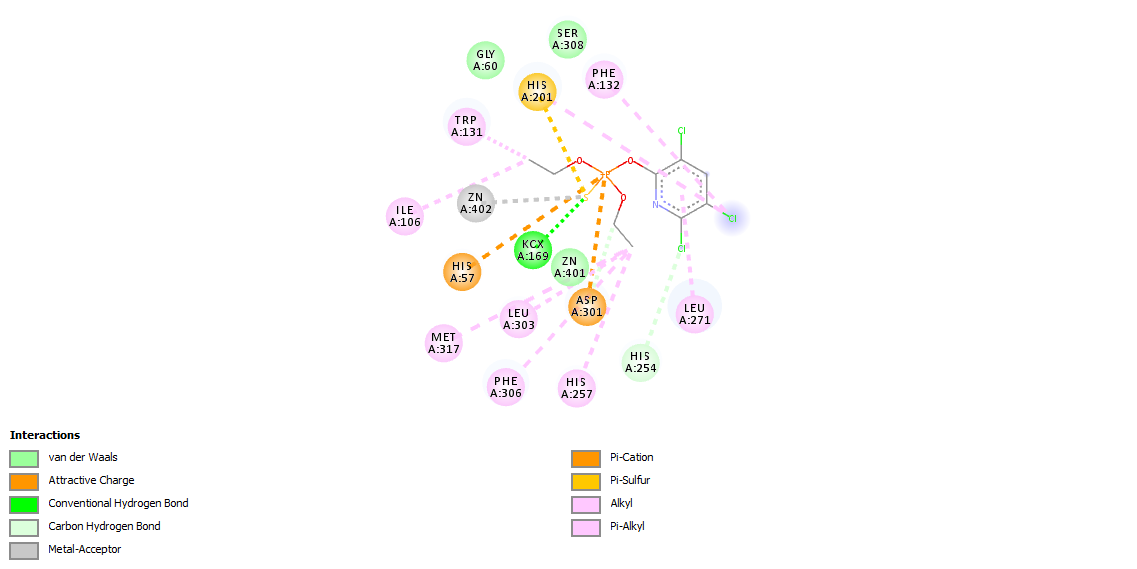


**Fig S1.** Molecular docking of chlorpyrifos pesticide in catalytic pocket of opdH enzyme, where conservred residues were interacted with pesticides.

**
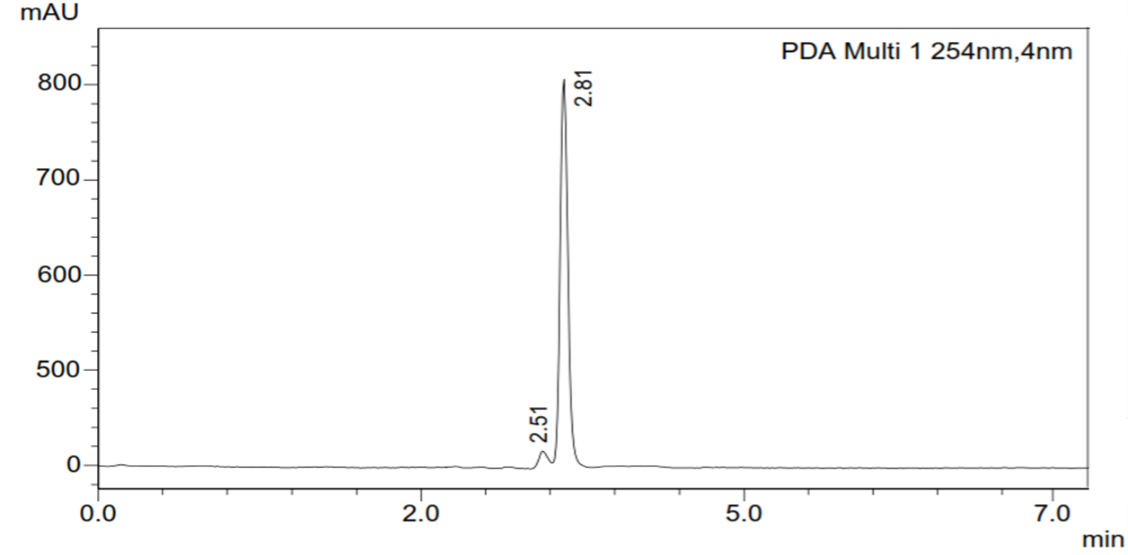
**

**Fig S2.** HPLC chromatogram of standard intermediate 3,5,6-Trichloro-2-pyridinol (TCP) of chlorpyrifos pesticide

**References**

Deng, S., Chen, Y., Wang, D., Shi, T., Wu, X., Ma, X., Li, X., Hua, R., Tang, X., Li, Q.X., 2015. Rapid biodegradation of organophosphorus pesticides by Stenotrophomonas sp: G1. J. Hazard. Mater. 297, 17–24. https://doi.org/10.1016/j.jhazmat.2015.04.052

Dhameliya, H.A., Mesara, S.N., Mali, H., Shah, C., Subramanian, R.B., 2020. Biochemical and Molecular Characterization of Lactic Acid Bacteria (LAB) Isolated from Fermented Pulses. Iran. J. Sci. Technol. Trans. A Sci. 44, 1279–1286. https://doi.org/10.1007/s40995-020-00934-z

K, T.S., 1999. Standard Methods of Biochemical Analysis. Kalyani Pub.

Tamura, K., Peterson, D., Peterson, N., Stecher, G., Nei, M., Kumar, S., 2011. MEGA5: molecular evolutionary genetics analysis using maximum likelihood, evolutionary distance, and maximum parsimony methods. Mol. Biol. Evol. 28, 2731–2739.

Yang, L., Zhao, Y., Zhang, B., Yang, C.-H., Zhang, X., 2005. Isolation and characterization of a chlorpyrifos and 3, 5, 6-trichloro-2-pyridinol degrading bacterium. FEMS Microbiol. Lett. 251, 67–73.
